# Synergistic immune protection of exosomal T-cell epitope vaccine and antibody-inducing vaccine against SARS-CoV-2 in highly humanized mice

**DOI:** 10.3389/fimmu.2026.1729444

**Published:** 2026-02-03

**Authors:** Anran Shen, Suyue Zhu, Min Li, Yu Zhao, Yandan Wu, Yue Zhang, Jiejie Zhang, Xuelian Han, Yuan Wang, Guangyu Zhao, Linli Lv, Qi Yin, Taotao Tang

**Affiliations:** 1 Institute of Nephrology, Zhongda Hospital, Medical School of Southeast University, Nanjing, China; 2 Department of Microbiology and Immunology, Medical School of Southeast University, Nanjing, China; 3 State Key Laboratory of Pathogen and Biosecurity, Academy of Military Medical Sciences, Beijing, China; 4 Laboratory of Advanced Biotechnology, Academy of Military Medical Sciences, Beijing, China

**Keywords:** antibody-inducing vaccine, combined immunization, exosomal vaccine, exosome, SARS-CoV-2, T-cell epitope vaccine

## Abstract

**Objective:**

To evaluate the synergistic immunological protection of exosomal T-cell epitope vaccine and antibody-inducing vaccine against SARS-CoV-2 in highly humanized mice.

**Methods:**

Red blood cell-derived exosomes were loaded with 27 CD8^+^ T-cell epitope peptides and 19 CD4^+^ T-cell epitope peptides followed by combined immunization with S1 protein vaccine of SARS-CoV-2 and adjuvant poly I:C in HLA-A2/DR1 double transgenic mice. After immunizations, splenocytes were assessed for epitope-specific T cell responses by intracellular cytokine staining and ELISPOT, and for functional T cell subset analysis through flow cytometry. Meanwhile, serum anti-S1 protein IgG and neutralizing antibodies were quantified via ELISA and BA.5 pseudovirus neutralization assay, respectively. Furthermore, viral challenge was performed after the combined immunization in HLA-A2/DR1/hACE2 triple transgenic mice, followed by viral load quantification, viral protein detection, and H&E staining in lungs.

**Results:**

The combined immunization i) increased the titers of S1 protein-specific IgG antibodies and neutralizing antibodies as well as Tfh cell frequency as compared to the S1 protein vaccine alone; ii) induced significantly more S1 protein-specific T cells and effector memory CD4^+^ T cells, and inhibited T cell exhaustion and regulatory T cell differentiation, compared to the exosomal T-cell epitope vaccine alone; iii) achieved the lowest pulmonary viral loads, inflammatory cell infiltration, and histopathological damage after SARS-CoV-2 infection.

**Conclusion:**

This study, for the first time, demonstrates the synergistic humoral and cellular immune responses and protective efficacy induced by the combined immunization of exosomal T-cell epitope vaccine and antibody-inducing vaccine, and provides preclinical evidence from highly humanized mice for optimizing next-generation SARS-CoV-2 vaccine protocols.

## Introduction

Infection of SARS-CoV-2 induces antigen-specific T cell immunity that critically determines disease progression and clinical outcomes (1). Asymptomatic or mild COVID-19 cases exhibit elevated neutralizing antibody titers coupled with robust CD4^+^ T cell and CD8^+^ T cell responses, whereas severe cases frequently demonstrate impaired coordination between cellular and humoral immunity, suggesting immune dysregulation as a key pathogenic mechanism (2). T-cell epitopes are distributed across SARS-CoV-2 proteome, not limited to the hypermutable receptor-binding domain (RBD) targeted by neutralizing antibodies, thus the T-cell immunity may be more likely to maintain cross-protective effects against viral variants than neutralizing antibodies. More importantly, coronavirus infections typically induce durable memory T cell responses, with protective memory persisting for 6–17 years as evidenced by longitudinal studies (2–5).

Therefore, the development of T-cell epitope vaccines has attracted increasing attention, and some vaccines have entered clinical trials, such as CoVepiT (NCT04885361, NCT04954469), IMP CoVac-1 (6, 7), PepGNP-SARSCoV2 (NCT04935801) (8), and UB-612 (9), and BNT162B4 (mRNA vaccine based on T-cell epitopes) (10). However, the T-cell epitope multipeptide vaccines are easily degraded *in vivo* and have weaker immunogenicity than the antibody-inducing vaccines. Another limitation is that they cannot directly neutralize viruses or block their entry into host cells, because they mainly eliminate infected cells by activating cellular immunity. Therefore, the T-cell epitope vaccines should be used in combination with antibody-inducing vaccines to exert synergistic effects, but the information from clinical combined use is still very limited. The key issues regarding their combinatorial use with antibody-inducing vaccines remain unclear, including unsubstantiated claims of T cell and B cell synergistic immune responses and uncertain protective efficacy advantages over mono-vaccine immunization.

To address these unsolved questions, the in-house generated HLA-A2/DR1 double transgenic mice and HLA-A2/DR1/hACE2 triple transgenic mice (also lacking murine MHC-I and MHC-II functions) were employed in this study as a preclinical platform for evaluating humanized vaccines. The mice only express human HLA-A0201 and HLA-DRA/HLA-DRB10101 molecules while knocking out murine H-2 class I and II molecules, thus can highly simulate the antigen presentation and T cell response process in human. In addition, the red blood cell (RBC)-derived exosomes were used here to carry T-cell epitope peptides using an in-house invented peptide-loading method, aiming to slow down the degradation rate of these multipeptides *in vivo* and adjust how these peptides are presented by antigen-presenting cells.

In our recent researches, 117 CD8^+^ T-cell epitopes restricted by 9 dominant HLA-A allotypes and 93 CD4^+^ T-cell epitopes restricted by 12 dominant HLA-DR allotypes have been identified from five main proteins of SARS-CoV-2 (11, 12); a method to loading multipeptides onto exosomes was invented without peptide modification and an exosomal vaccine by anchoring T-cell epitope peptides of SARS-CoV-2 onto exosomal membrane has been generated and induced robust CD8^+^ T cell responses in HLA-A2/DR1 transgenic mice (13). Here, 19 HLA-DR1-restricted and 27 HLA-A2-restricted epitopes were selected to formulate a peptide cocktail, generate an exosomal T-cell epitope vaccine, and perform a combined immunization with recombinant S1 protein of SARS-CoV-2 in HLA-A2/DR1 transgenic mice. The synergistic immunological responses were demonstrated by the enhanced cellular and humoral immune responses compared to mono-vaccine immunization. Furthermore, the viral challenge was performed after the combined immunization in the HLA-A2/DR1/hACE2 transgenic mice, demonstrating the synergistic protective efficacy against virus infection, including the significant reductions in viral loads, pulmonary inflammatory cell infiltration, and histopathological damage, as compared with mono-vaccine immunization. Our data provide preclinical evidence from highly humanized mice for the synergistic ability of exosomal T-cell epitope vaccine with antibody-inducing vaccine in enhancing protective immunity against SARS-CoV-2 infection.

## Materials and methods

### Ethical approval and human blood samples

After red blood cells were separated, the white blood cell filter trays were collected from the Blood Component Preparation Section of Nanjing Red Cross Blood Center from October 2023 to April 2024. These clinical specimens from healthy blood donors were obtained through routine erythrocyte separation procedures without additional donor-specific interventions. The collection and use of blood samples were approved by the Clinical Ethics Committee of Nanjing Red Cross Blood Center later (January, 2022; ref: 2022-015-01). The informed consent for each blood donor was obtained from Nanjing Red Cross Blood Center. The residual red blood cells in the white blood cell filter trays were immediately separated using Ficoll-Paque density-gradient centrifugation and used directly for exosome preparation.

### Mice

Two types of HLA transgenic C57BL/6 mice were used in this study. The HLA-A2/DR1 mice (HLA-A0201^+/+^/DRA^+/+^/DRB10101^+/+^/β2m^−/−^/I-Aβ^−/−^) and HLA-A2/DR1/hACE2 mice (HLA-A0201^+/+^/DRA^+/+^/DRB10101^+/+^/β2m^–/–^/I-Aβ^–/–^/hACE2^+/+^) were obtained from the State Key Laboratory of Pathogen and Biosecurity, Academy of Military Medical Sciences (Beijing, China). These female mice were housed in the specific pathogen-free Animal Centre of Academy of Military Medical Sciences and used at 8 weeks of age. All animal welfare and experimental procedures were conducted in strict accordance with the Guide for the Care and Use of Laboratory Animals (Ministry of Science and Technology of China, 2006) and were approved by the Animal Ethics Committee of Academy of Military Medical Sciences (Permit Number: IACUC-IME-2022-004).

### Peptides, recombinant S1 protein, HEK-293T-ACE2 cells and SARS-CoV-2 BA.5 variant

The peptides were synthesized by Nanjing GenScript Biotech Co., Ltd. (Nanjing, China) and demonstrated a purity exceeding 95% through HPLC and mass spectrometry analysis. The lyophilized peptides were reconstituted in a DMSO-PBS solution to prepare stock solutions with concentrations of 5 mg/mL for subsequent cellular functional experiments. The recombinant S1 protein was purchased from Sino Biological, Inc. (40591-V08H46, Beijing, China). It corresponds to the spike protein S1 subunit of SARS-CoV-2 (BA.4/BA.5/BA.5.2 variants), with the first 9 amino acid residues of its signal peptide deleted. Comprising 676 amino acids (Met1-Arg685), this protein predominantly contains a receptor-binding domain (RBD) and a C-terminal His tag. Prior to lyophilization, the protein is formulated in sterile PBS supplemented with 5%–8% trehalose, mannitol, and 0.01% Tween 80 as protectants. HEK-293T-ACE2 cells were from the State Key Laboratory of Pathogen and Biosecurity, Academy of Military Medical Sciences (Beijing, China). SARS-CoV-2 BA.5 variant was from the Laboratory of Advanced Biotechnology, Academy of Military Medical Sciences (Beijing, China).

### Preparation of RBC-derived exosomes

RBC-derived exosomes were isolated using our established protocol (13). Briefly, white blood cell filter trays were back-flushed with PBS to retrieve fresh blood cells. The residual RBCs were immediately separated using Ficoll-Paque density-gradient centrifugation, followed by two washing cycles in PBS (3,000 × g for 10 min at 4°C. The washed RBC were resuspended in PBS at 1:50 (v/v) and incubated under constant agitation (100 rpm) at 4°C for 48 h. Then, the suspension underwent sequential centrifugation: 3,000 × g for 20 min at 4°C (pellet discarded); 13,500 × g for 30 min at 4°C (large extracellular vesicles removed). The supernatant was filtered through 0.22 μm PVDF membranes (Millipore) followed by ultracentrifugation at 200,000×g for 2 h at 4°C (XPN-Optima 100 ultracentrifuge, Beckman Coulter, CA, USA). The final exosomal pellet was resuspended in 100 μL PBS and preserved at -80°C for further identification and usage.

### Preparation and purification of peptides-PEG-lipid-exosomes

19 HLA-DR1-restricted CD4^+^ T-cell epitope peptides and 27 HLA-A2-restricted CD8^+^ T-cell epitope peptides from five main proteins of SARS-CoV-2 were initially solubilized in sterile saline or minimal DMSO (<0.1% final concentration, v/v) and mixed together. **Table 1** listed the detailed information of 46 epitopes. The peptide cocktail was subsequently diluted with sterile saline to achieve a working concentration of 5 μg/μL, aliquoted to prevent freeze-thaw cycles, and preserved at -80°C until use. The preparation of peptides-PEG-lipid-exosomes was conducted as described (13). Briefly, DMPE-PEG-NHS (1,2-Dimyristoyl-sn-glycero-3-phosphoethanolamine-PEG-NHS; MW:4257.98, customized in Ponsure Biological, Shanghai) and the peptide cocktail were incubated at a 1:3 molar ratio with 1% TEA (triethylamine; Aladdin, Shanghai) overnight at room temperature (RT) to form the peptides-PEG-DMPE complex. Then, DMSO concentration was diluted below 1% by HEPES buffer, and DSPE-PEG (1,2-Distearoyl-sn-glycero-3-phosphoethanolamine-PEG; MW: 2805.497, Ponsure Biological) was added at a 1:1 molar ratio to peptides-DMPE-PEG-NHS complex and further incubated for 15 min at 60°C to form micelles. The free peptides were removed, and the protein levels in the resultant peptides-PEG-micelles were quantified using the BCA assay kit and stored at 4°C for use in the following 2 weeks. The reserved peptides-PEG-micelles were reshuffled by ultrasonicated to reduce the micelle size, and followed by co-incubation with RBC-derived exosomes at a 1:1 protein ratio for 2 h at 40°C. The solution was cooled to 4°C, then immediately purified using a fast protein liquid chromatography (FPLC) system (Pharmacia Fine Chemicals, Uppsala, Sweden) with the SXK16/40 Smartarose CL-4B column (SEC0081, Smart-Lifesciences Biotechnology Co. Ltd., Changzhou, China). The second peak fractions were collected, ultrafiltration-concentrated in saline, and designated as the peptides-PEG-lipid-exosomes by western blot assay.

**Table 1:** Detailed information of 46 T-cell epitopes used to immunize mice.

19 HLA-DR-restricted CD4^+^ T-cell epitopes	Sequence	Protein	27 HLA-A2-restricted CD8^+^ T-cell epitopes	Sequence	Protein
Z678	RLCAYCCNIVNVSLVK	E	A1	FLAFVVFLL	E
Z723	SFYVYSRVKNLNSSRV	E	A3	VLLFLAFVV	E
Z469	MWSFNPETNILLNVPL	M	A4	FLLVTLAIL	E
Z388	LPKEITVATSRTLSYY	M	A5	RLCAYCCNIV	E
Z249	ICLLQFAYANRNRFLY	M	B1	GLMWLSYFI	M
Z31	AVYRINWITGGIAIAM	M	B3	FVLAAVYRI	M
Z702	SASAFFGMSRIGMEVT	N	B4	FLFLTWICLL	M
Z328	KQQTVTLLPAADLDDF	N	B6	TLACFVLAAV	M
Z82	DGIIWVATEGALNTPK	N	C1	LLLDRLNQL	N
Z547	NTASWFTALTQHGKED	N	C2	GMSRIGMEV	N
Z735	SGTWLTYTGAIKLDDK	N	C3	WLTYTGAIKL	N
Z790	TATKAYNVTQAFGRRG	N	D2	FIAGLIAIV	S
Z92	DGVPFVVSTGYHFREL	RdRp	D5	KLNDLCFTNV	S
Z183	FKSVLYYQNNVFMSEA	RdRp	D6	RLDKVEAEV	S
Z532	NPDILRVYANLGERVR	RdRp	D7	VLNDILSRL	S
Z454	MPNMLRIMASLVLARK	RdRp	D11	VVFLHVTYV	S
Z885	VNFNFNGLTGTGVLTE	S	D12	FVFLVLLPLV	S
Z638	QSIIAYTMSLGAENSV	S	D13	MIAQYTSAL	S
Z814	TQDLFLPFFSNVTWFH	S	R4	AMRNAGIVGV	RdRp
			R5	SLAIDAYPL	RdRp
			R6	NLLKDCPAV	RdRp
			R8	FVNEFYAYL	RdRp
			R9	ILHCANFNV	RdRp
			R10	KIFVDGVPFV	RdRp
			R13	NMLRIMASL	RdRp
			R14	RLANECAQV	RdRp
			R15	QLLFVVEVV	RdRp

### Combined immunization of exosomal vaccine and S1 protein vaccine in HLA-A2/DR1 transgenic mice

Twenty 8-week-old female HLA-A2/DR1 transgenic mice were randomly allocated into four experimental cohorts (n = 5). For the adjuvant/exosomes group: 400 μL empty exosomes and 100 μg poly I:C (Invivogen, vac-pic, Carlsbad, CA, USA) were injected per mouse per time point. First, 100 μg mixture of poly I:C and empty exosomes was diluted with sterile saline to 400 μL, and administered subcutaneously (s.c) to bilateral inguinal, caudal base, and cervical regions. Then, another 100 μg of the mixture was diluted to 200 μL and administered intramuscularly (i.m) to bilateral quadriceps femoris muscle sites. For the S1 protein vaccine group: 200 μL of the solution containing 200 μg BA.5 recombinant S1 protein and 100 μg poly I:C in sterile saline was administered i.m into bilateral quadriceps. For the exosomal vaccine group, 400 μL of the solution containing peptides-PEG-lipid-exosomes (containing 230 μg peptides, 5.0 μg/peptide/mouse/time point) and 100 μg poly I:C in sterile saline was administered s.c into bilateral inguinal regions, caudal base, and cervical triangle. For the combined immunization group, the peptides-PEG-lipid-exosomes peptide (400 μL) mixed with 50 μg poly I:C was injected s.c into four sites (bilateral inguinal, caudal base, and cervical regions), meanwhile the S1 protein (200 μg) mixed with poly I:C (50 μg) was injected i.m into bilateral quadriceps of the mouse. While researchers conducting animal experiments were informed of the animal grouping, personnel responsible for detecting T-cell responses remained unaware of such grouping details. This arrangement minimized subjective bias during the evaluation process and guaranteed the objectivity of the results.

On day 0, each group was injected as the primary immunization. After that, booster immunizations were administered on day 7 and 21 with same dose as primary immunization. On day 28, the mice were first anesthetized with 2% isofurane (Shanghai Abbott Pharmaceutical Co. Ltd.) and then euthanized via CO_2_ inhalation. The spleen cells were extracted for further tests.

### Detection of epitope-specific T cell response using intracellular cytokine staining

The 46 epitope peptides used to immunize mice were grouped into 10 peptide pools according to their chemical characteristics and derived protein (**Table 2**). Mice were euthanized on the 7th day post-third immunization, where splenocytes from immunized mice were prepared into single-cell suspensions and seeded in 48-well cell culture plates at 2×10^6^ cells/well, 13 wells/sample. Each experimental well was incubated with one peptide pool (20 μg/mL/peptide, totally ten wells), while the remaining three control wells were incubated with phytohemagglutinin (PHA, 2030411, Dakewe, Shenzhen, China, 10 μg/mL), irrelevant peptide (HLA-A2-restricted AFP_158–166_ or HLA-DR1-restricted AFP_421-436_, 20 μg/mL), and no peptide (spleen cell alone), respectively, for 16 hours in serum-free cell culture medium (Dakewe, 6115012) at 37°C and 5% CO_2_. Subsequently, a BFA/monensin mixture (Biolegend, 420701; 420601) was added to the cells for another 6-hour culture. Then, the cells were harvested, washed, blocked with anti-mouse CD16/CD32 (Miltenyi Biotec, 130-092-575) for 20 minutes at 4°C, and stained with FITC-anti-mouse CD3 (eBioscience, 11-0031-85, San Diego, CA, USA) and PE-anti-mouse CD4 antibodies (eBioscience, 12-0041-83) or PE-anti-mouse CD8a antibodies (eBioscience, 12-0081-83) for 30 minutes at 4°C. After washing, the cells were fixed and permeabilized using Fix&Perm kit (Multi Sciences, GAS005) following the protocol and further incubated with APC-anti-mouse IFN-γ (BD Bioscience, 554413) for another 30 min at 4°C followed by flow cytometry (Guava^®^ easyCyteTM HT). The frequencies of IFN-γ^+^ cells in the CD3^+^/CD4^+^ populations or CD3^+^/CD8^+^ populations were calculated.

**Table 2:** The peptide pools of 46 T-cell epitopes used in ICS flow cytometry and ELISpot assay for the detection of epitope-specific T cells.

Peptide pools used in ICS flow cytometry			Peptide pools used in ELISpot assay		
Pool	Epitopes	Protein	Pool	Epitopes	Protein
CD4-E	Z678, Z723	E	Pool-1	Z678, Z723, A1, A3, A4, A5	E
CD4-M	Z469, Z388, Z249, Z31	M	Pool-2	Z388, Z249, Z31, B3	M
CD4-N	Z702, Z328, Z82, Z547, Z735, Z790	N	Pool-3	Z469, B1, B4, B6	M
CD4-S	Z885, Z638, Z814	S	Pool-4	Z328, Z82, Z547	N
CD4-R	Z92, Z183, Z532, Z454,	R	Pool-5	Z702, Z735, Z790, C1, C2, C3	N
CD8-E	A1, A3, A4, A5	E	Pool-6	Z183, Z454, Z532, R4, R9, R10, R13	R
CD8-M	B1, B3, B4, B6	M	Pool-7	Z92, R5, R6, R8, R14, R15	R
CD8-N	C1, C2, C3	N	Pool-8	Z885, Z638, Z814, D5, D6	S
CD8-S	D2, D5, D6, D7, D11, D12, D13	S	Pool-9	D2, D7, D11, D12, D13	S
CD8-R	R4, R5, R6, R8, R9, R10, R13, R14, R15	R			

### Detection of epitope-specific T cell response via ELISpot assay

For ELISpot assay, the 46 epitope peptides used to immunize mice were grouped into 9 peptide pools according to their chemical characteristics and derived protein (**Table 2**). Spleen cells from each mouse were seeded into 12 wells in 96-well ELISpot plates (2×10^5^ cells/100 μL/well) (Merck & Millipore, M8IPS4510, Billerica, MA, USA) pre-coated with mouse IFN-γ capture antibody (BD Pharmingen, 551873, 1:200 dilution). Each experiment well was co-cultured with one peptide pool (2 μg/peptide/well, totally 9 wells) for 20 hours in serum-free cell culture medium (Dakewe, 6115012, Shenzhen, China) at 5% CO_2_ and 37°C, while the remaining three wells were incubated with PHA (2.5 μg/well), irrelative peptides (AFP_158–166_ and AFP_421-436_, 2 μg/peptide/well) and spleen cells alone, respectively. After incubation, spots were developed using a mouse IFN-γ detection antibody (BD Pharmingen, 551873, 1:250 dilution), streptavidin-HRP (BD Pharmingen, 557630, 1:100 dilution), and an AEC substrate set (BD Pharmingen, 551951, 1:50 dilution), following the manufacturer’s protocol. Spot-forming units (SFUs) were imaged and counted using a Mabtech IRISTM ELISPOT & FluoroSpot Reader (Mabtech, Sweden). SFUs in each experimental well were determined by subtracting the spot count in the negative control well from the count in the experimental well. If the spot count in the experimental well was lower than that in the negative control, the SFUs in the experimental well were recorded as zero. The total SFUs per 2 × 10^5^ spleen cells stimulated by all peptide pools were calculated by summing the SFUs across all experimental wells. Additionally, SFUs per 2 × 10^5^ spleen cells stimulated by the peptides from each SARS-CoV-2 protein were computed based on the peptide pools corresponding to each protein.

### Analysis of T cell functional subsets via multicolor flow cytometry

Splenocytes (4×10^6^ cells/mouse) were subjected to centrifugation (300 × g, 4 min, 25 °C) and resuspended in 350 μL PBS supplemented with 35 μL Fc block (anti-mouse CD16/CD32, Miltenyi Biotec, 130-092-575), then aliquoted into 7 staining tubes (6 experimental, 1 FMO control; 55 μL/tube) followed by incubation for 10 min at 4 °C. Multicolor immunostaining was performed using pre-titrated antibody cocktails with 6 marker panels. Tube 1: FITC-anti-CD3ϵ (eBioscience, 11-0031-85, clone 145-2C11), PE-anti-CD4 (eBioscience, 12-0041-83, clone GK1.5), APC-anti-CD62L (eBioscience, 17-0621-82, clone MEL-14), PE-Cy7-anti-CD44 (eBioscience, 25-0441-82, clone IM7); Tube 2: FITC-anti-CD3ϵ, PE-anti-CD8α (eBioscience, 12-0081-83, clone 53-6.7), APC-anti-CD62L, PE-Cy7-anti-CD44; Tube 3: FITC-anti-CD3ϵ, PE-anti-CD4, PE-Cy7-anti-CD25 (eBioscience, 25-0251-82, clone PC61), APC-anti-Foxp3 (Biolegend, 126408, clone FJK-16s); Tube 4: FITC-anti-CD3ϵ, PE-anti-CD8α, APC-anti-PD-1 (eBioscience, 17-9985-82, clone J43); Tube 5: FITC-anti-CD3ϵ, PE-anti-CD4, APC-anti-PD-1; Tube 6: FITC-anti-CD3ϵ, PE-anti-CD4, PE-Cy7-anti-CD185 (eBioscience, 25-7185-82, clone SPRCL5). After 30 min incubation at 4°C, cells were washed for three times with PBS and then analyzed by Flow Cytometer (Guava^®^ easyCyteTM HT).

### Detection of serum S1 protein-specific IgG antibodies via ELISA

The 96-well plates were coated with recombinant S1 protein of SARS-CoV-2 BA.5 strain (50 μL/well of 20 μg/mL) in carbonate-bicarbonate buffer (pH 9.6) overnight at 4°C, then washed, and blocked with 2% BSA-PBS for 90 min at 25°C. The serum sample was serially diluted, and incubated in the pre-coated wells (100 μL/well) for 2 hours, then incubated with HRP-conjugated goat anti-mouse IgG (Solarbio, SE132, Beijing, China) for 30 minutes first and TMB solution (Solarbio, PR1210) for 15 minutes later. After each incubation step, the wells were rigorously washed to reduce background interference. The reaction was terminated with a stop solution (Solarbio, C1058), and antibody levels were quantified as titers by measuring absorbance values (OD450/630) using a SpectraMax^®^ i3x Multi-Mode Reader (Molecular Devices, CA, USA).

### Detection of neutralizing antibodies using SARS-CoV-2 BA.5 pseudovirus

SARS-CoV-2-Fluc BA.5 pseudoviruses (Vazyme Biotech, DD1776, Nanjing, China) were diluted to the stock concentration of 2×10^4^ TCID_50_/mL and seeded into a 96-well cell culture plate (50 μL/well). Heat-inactivated mouse serum (150 μL) was initially diluted 6.67-fold in viral maintenance medium (VMM; 850 μL 2% FBS-DMEM), then serially diluted across 8 test wells (including initial concentration) using 3-fold dilution steps. Control wells included (1): virus control (100 μL VMM) and (2) cell control (100 μL VMM + 50 μL complete medium with 10% FBS/1% penicillin-streptomycin). The pseudoviruses in experimental wells were then neutralized with serially diluted murine serum (100 μL/well) at 37 °C for 1 h (final serum dilution factor 10 in initial well). After that, HEK293T/hACE2 cells (stably expressing human ACE2, Vazyme Biotech, DD1401) were seeded into each well (2×10^4^ cells/50 μL/well) followed by co-culture for 48 h. Then, luminescent readout was performed using Bio-Lite™ Luciferase Assay Reagent (100 μL/well; Thermo Fisher) after 5 min dark adaptation, with relative light units (RLU) quantified at 570 nm using a SpectraMax^®^ i3x system (Molecular Devices).

### Combined immunization of exosomal vaccine with S1 protein vaccine followed by viral challenge in HLA-A2/DR1/hACE2 mice

Twenty 8-week-old HLA-A2/DR1/hACE2 transgenic female mice were randomized into four cohorts (n=5/group), with primary immunization performed on day 0, followed by booster immunizations on day 7 and day 21 as described above. The preparation, dose and injection regimens of S1 protein vaccine and exosomal vaccine, and the experimental groups were identical to the combined immunization experiment in HLA-A2/DR1 transgenic mice. On day 28 (7 days post-final boost), cohorts were transferred to ABSL-3 containment of the Laboratory of Advanced Biotechnology, Academy of Military Medical Sciences (Beijing, China), and challenged intranasally with 10,000 TCID_50_ of authentic SARS-CoV-2 BA.5 (50 μL/mouse). Viral loads were quantified via qRT-PCR from homogenized lung tissues on day 3, while histopathological analysis employed H&E staining and immunohistochemical staining.

### Detection of viral load in lungs of HLA-A2/DR1/hACE2 mice

Three days after viral challenge, the lungs of HLA-A2/DR1/hACE2 transgenic mice were collected and pulverized using a Freezer/Mill Cryogenic Grinder. Then, the total RNA of lung was extracted by TRIZOL method. Finally, RNA was reverse-transcribed and the E subgenomic mRNA of SARS-CoV-2 was quantified with qRT-PCR using Premix Ex Taq (Probe qPCR) kit (Takara, RR390Q, Tokyo, Japan) and a StepOnePlus™ Real-Time Detection System. The Primer-F (CGATCTCTTGTAGATCTGTTCTC), Primer-R (ATATTGCAGCAGTACGCACACA) and Probe (FAM-ACACTAGC-CATCCTTACTGC GCTTCG-BBQ) were used.

### Hematoxylin-Eosin staining and immunohistochemical staining

Mice were euthanized. Pulmonary tissues were collected and fixed in 4% PFA for 24h, processed through graded ethanol series, and paraffin-embedded. Sequential 5-μm sections were dewaxed and rehydrated for hematoxylin-eosin (H&E) staining and microscopic imaging following standard histopathological protocols. To further detect the viral load in lungs, the nucleocapsid protein of SARS-CoV-2 was detected by immunohistochemical staining (IHC), using rabbit-anti-nucleocapsid antibody (Abcam plc, ab281296, clone HL344, Cambridge, England), HRP-conjugated goat-anti-rabbit IgG (H+L) (Beyotime Biotech., A0208, Shanghai, China), and DAB substrate (Beijing Zhong Shan- Golden Bridge Biotech., Beijing, China).

### Statistic

Statistical analyses and plot drawing were conducted using GraphPad Prism v.9.1.0 and R software. Mann–Whitney (nonparametric) test was used to test the differences between two groups, while Kruskal–Wallis H test was used to test the differences between multiple groups. The p-value less than 0.05 was considered significant.

## Results

### Characterization of RBC-derived exosomes and preparation of exosomal T-cell epitope vaccine

**Figure 1:**
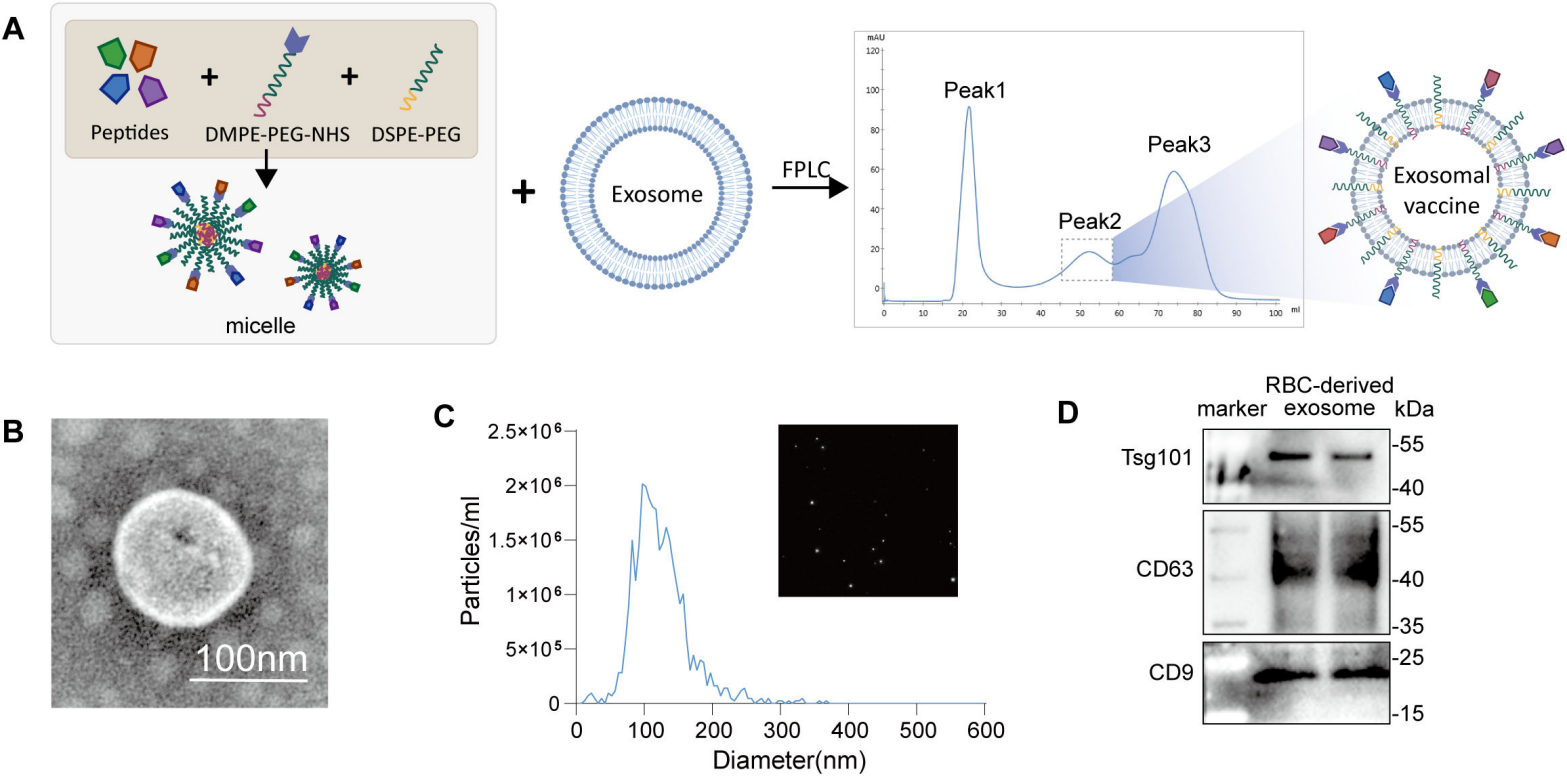
Characterization of RBC-derived exosome and preparation of exosomal T-cell epitope vaccine. **(A)** The diagram of exosomal T-cell epitope vaccine preparation; **(B)** Transmission electron microscope showing morphology of RBC-derived exosomes; **(C)** NTA showing the diameter of RBC-derived exosomes; **(D)** Western Blotting analysis of ALIX, CD63, CD9 markers in RBC-derived exosomes.

The RBC-derived exosomes and exosomal T-cell epitope vaccine were generated according to the procedure we established previously (13). A peptide cocktail was prepared, including 27 HLA-A2-restricted and 19 HLA-DR1-restricted T-cell epitopes, which were previously identified from the spike glycoprotein (S), envelope protein (E), membrane protein (M), nucleocapsid protein (N), and RNA-dependent RNA polymerase (RdRp) of SARS-CoV-2 (11, 12). The NHS group-conjugated lipid-PEG complexes (DMPE-PEG-NHS) were generated according to the manufacturer’s instructions. The NHS group can form covalent bonds with the COOH groups of peptide or protein. This enables DMPE (lipid) to complex with any peptide or protein of interest. Thus, the peptides were conjugated with DMPE-PEG-NHS to form peptides-PEG-DMPE complexes and further mixed with DSPE-PEG to form peptide-PEG-micelles with a loading efficiency of around 91%, and finally were allowed to insert into the lipid bilayers of the exosomes by self-assembly and structure relaxation caused by high temperature. The resulting peptides-PEG-lipid-exosomes solution was instantly purified using size-exclusion chromatography (**Figure 1A**). The FPLC profile showed three major peaks. The second peak represented the peptides-PEG-lipid-exosome complex (exosomal T-cell epitope vaccine), because this mixture contained high-levels of peptides, DMPE-PEG, hydrolytic NHS, and exosome (CD63 inter), and displayed typical exosome morphology, as determined by Western blotting, the liquid nuclear magnetic resonance (NMR) test and transmission electron microscopy [13]. RBC-derived exosomes were characterized as typical round shape vesicle (**Figure 1B**) with an average diameter of 125 nm (**Figure 1C**) and exosome markers expression (**Figure 1D**).

### Combined immunization of exosomal T-cell epitope vaccine and recombinant S1 protein vaccine elicits more robust T cell responses in HLA-A2/DR1 transgenic mice

**Figure 2:**
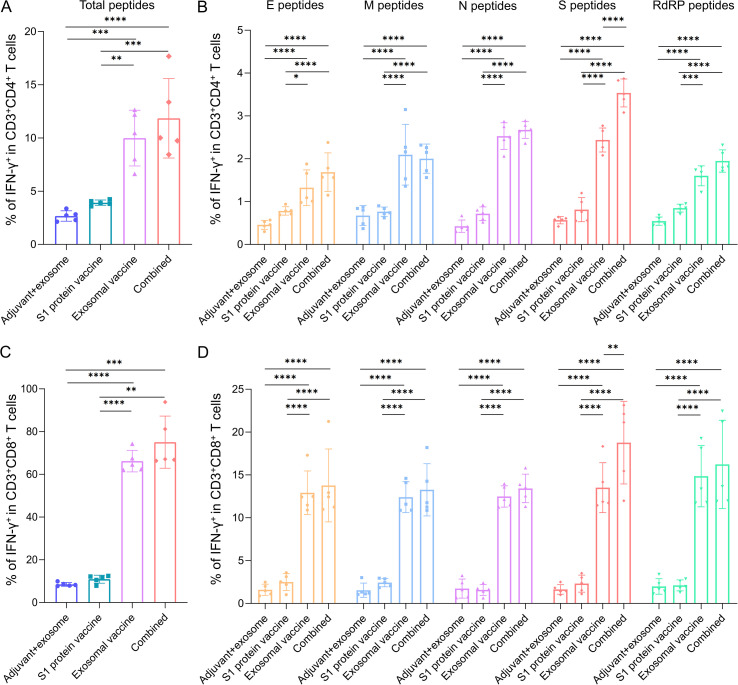
Frequency profiles of epitope-specific CD4^+^ T cells and CD8^+^ T cells in each immunization group as detected by ICS and flow cytometry. On day 28 post-immunization, splenocytes from each mouse in each immunization group (containing adjuvant poly I:C) were *ex vivo* co-cultured with 10 peptide pools (46 T-cell epitopes derived from E, M, N, S, and RdRp proteins and used to immunize mice) for 22 hours followed by IFN-γ ICS assays. **(A)** The frequencies of CD4^+^ T cells reactive to the total 46 T-cell epitope peptides in each group; **(B)** The frequencies of CD4^+^ T cells reactive to the T-cell epitope peptides derived from each protein in each group; **(C)** The frequencies of CD8^+^ T cells reactive to the total 46 T-cell epitope peptides in each group; **(D)** The frequencies of CD8^+^ T cells reactive to the T-cell epitope peptides derived from each protein in each group. Five HLA-A2/DR1 transgenic mice per group. Mann-Whitney test and Kruskal–Wallis H test were used. ****p<0.0001; ***: p<0.001; **p<0.01; *p<0.05.

In our previous studies, multiple immunization experiments were performed using the HLA-A2/DR1double-transgenic mice to induce T-cell response, with the immunogens including T-cell epitope peptide vaccines, PLGA nanoparticle-peptide vaccines, and exosomal peptide vaccines of SARS-CoV-2 (11–13). Based on these results, the immunization and detection protocol for the present study were designed. After immunization on day 0, day 7, and day 21, spleen cells were collected from each mouse in each group on day 28. The 46 epitope peptides used to immunize mice were classified into 10 peptide pools, and co-cultured *ex vivo* with the spleen cells followed by intracellular cytokine staining (ICS) to quantify the frequencies of IFN-γ^+^ cells in the CD3^+^/CD4^+^ populations or CD3^+^/CD8^+^ populations. As compared to the adjuvant + exosome control group, the S1 protein vaccine group displayed a similar frequency of IFN-γ^+^/CD4^+^ T cells, but exosomal vaccine group and the combined immunization group of S1 protein vaccine and exosomal vaccine exhibited 3.7-fold and 4.4-fold higher frequencies of IFN-γ^+^/CD4^+^ T cells, respectively (**Figures 2A, B**). Similarly, the exosomal vaccine group and combined immunization group demonstrated 7.8-fold and 8.8-fold higher frequencies of IFN-γ^+^/CD8^+^ T cells, respectively, as compared to the adjuvant+ exosome control group, while S1 protein vaccine did not elicit significant CD8^+^ T cell response (**Figures 2C, D**).

For each protein of SARS-CoV-2, the T cell responses also displayed a trend similar to the overall antigen-specific T cell responses in the four groups. Of note is that combined group elicited significantly stronger CD4^+^ T cell and CD8^+^ T cell responses specific to S protein rather than other proteins than the exosomal T-cell epitope vaccine group (p<0.01, **Figures 2B, D**), but no significant differences on S protein or other protein-specific CD4^+^ T cells and CD8^+^ T cells were found between S1 protein vaccine group and the adjuvant+ exosome control group. These data suggest that S1 protein vaccine can help the exosomal T-cell epitope vaccine elicit S protein-specific T cell responses, implying the synergistic enhancement of S protein-directed immunity through combined immunization of S1 protein and T-cell epitope peptides. The flow cytometric plots of each mouse are shown in **Supplementary Figure S1** (CD4^+^ T cells) and **Supplementary Figure S2** (CD8^+^ T cells).

**Figure 3:**
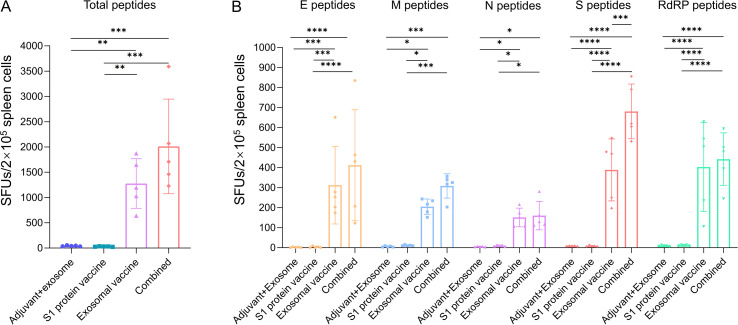
Count profiles of epitope-specific T cells in each immunization group as detected by ELISpot assay. On day 28 post-immunization, splenocytes from each mouse in each immunization group (containing adjuvant poly I:C) were *ex vivo* co-cultured with 9 peptide pools (46 T-cell epitopes derived from E, M, N, S, and RdRp proteins and used to immunize mice) for 20 hours followed by IFN-γ ELISpot assays. **(A)** The counts (SFUs/2×10^5^ splenocytes) of T cells reactive to the total 46 T-cell epitope peptides in each group; **(B)** The counts (SFUs/2×10^5^ splenocytes) of T cells reactive to the T-cell epitope peptides derived from each protein in each group. Five HLA-A2/DR1 transgenic mice per group. Mann-Whitney test and Kruskal–Wallis H test were used. ****p<0.0001; ***p<0.001; **p<0.01; *p<0.05.

Furthermore, the IFN-γ ELISpot assays were also performed to evaluate the epitope-specific T cell responses. The 46 epitope peptides used to immunize mice were classified into 9 peptide pools, and co-cultured *ex vivo* with the spleen cells followed by IFN-γ ELISpot assays to quantify the numbers of T cells reactive to the epitope peptides. As compared to the adjuvant+ exosome control group, the S1 protein vaccine group did not enhance the number of reactive T cells, but exosomal vaccine group and the combined immunization group exhibited 30.1-fold and 47.4-fold increased SFUs of IFN-γ-secreting cells, respectively, without significant difference between the two groups (**Figure 3A**). **Figure 3B** displayed the SFUs of T cells reactive to the individual protein-derived peptide pools, demonstrating a trend similar to the overall antigen-specific T cell responses in the four groups. The ELISpot dot plots of each mouse are presented in **Supplementary Figure S3**.

**Figure 4:**
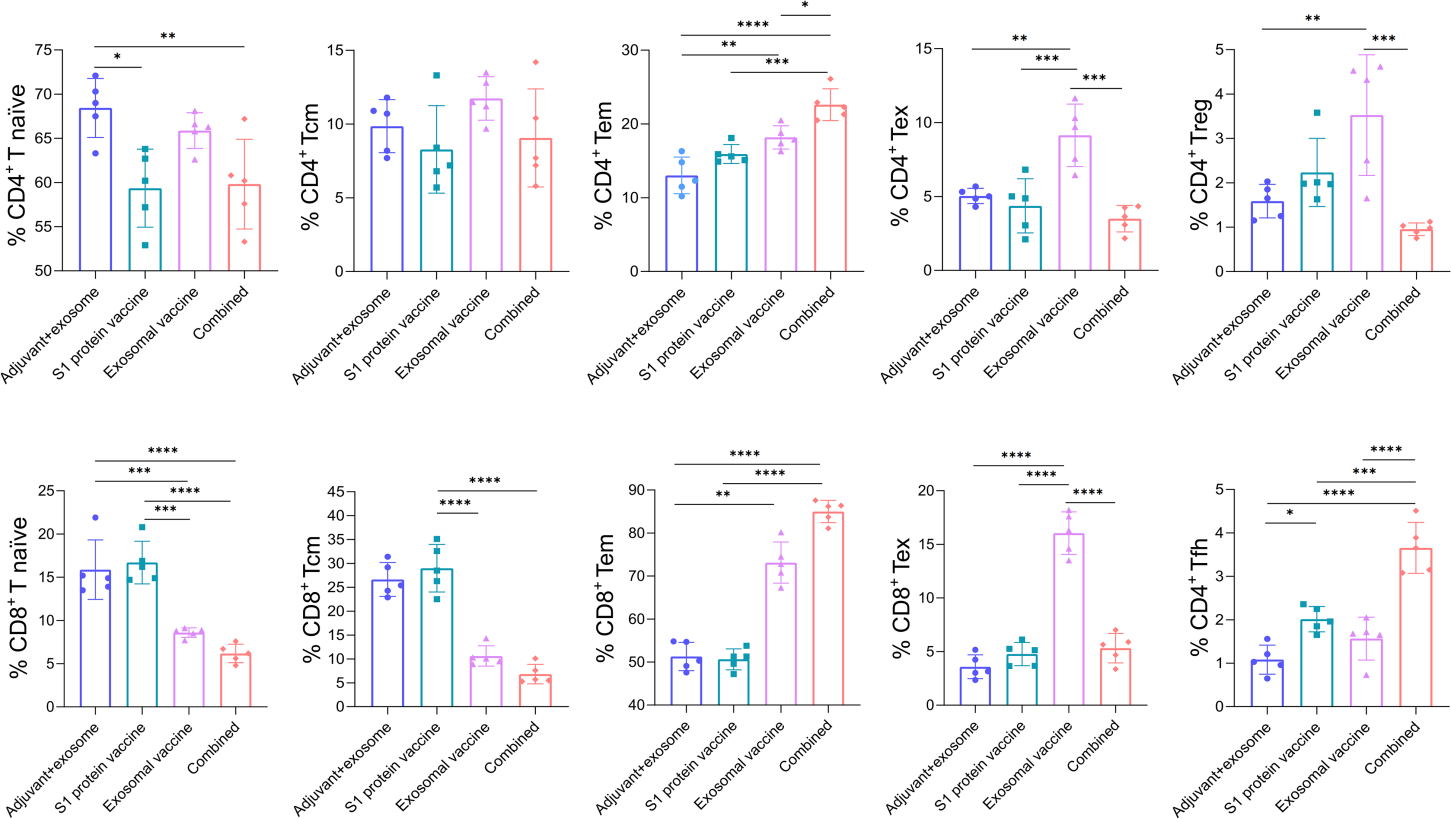
Frequency profiles of functional T cell subsets in each immunization group as detected by multicolor flow cytometry. On day 28 post-immunization, splenocytes from each mouse in each immunization group (containing adjuvant poly I:C) were stained with several groups of fluoroscent-labeled antibodies followed by functional phenotype analysis through multicolor flow cytometry. T naive: naive T cells (CD62L^+^/CD44^-^); Tcm: central memory T cells (CD62L^+^/CD44^+^); Tem: effector memory T cells (CD62L^-^/CD44^+^); Tex: exhausted T cells (PD-1^+^); Treg: regulatory T cells (FoxP3^+^/CD4^+^); Tfh: follicular helper T cells (CD185^+^/CD4^+^). Five HLA-A2/DR1 transgenic mice per group. Mann-Whitney test and Kruskal–Wallis H test were used. ****p<0.0001; ***p<0.001; **p<0.01; *p<0.05.

**Figure 5:**
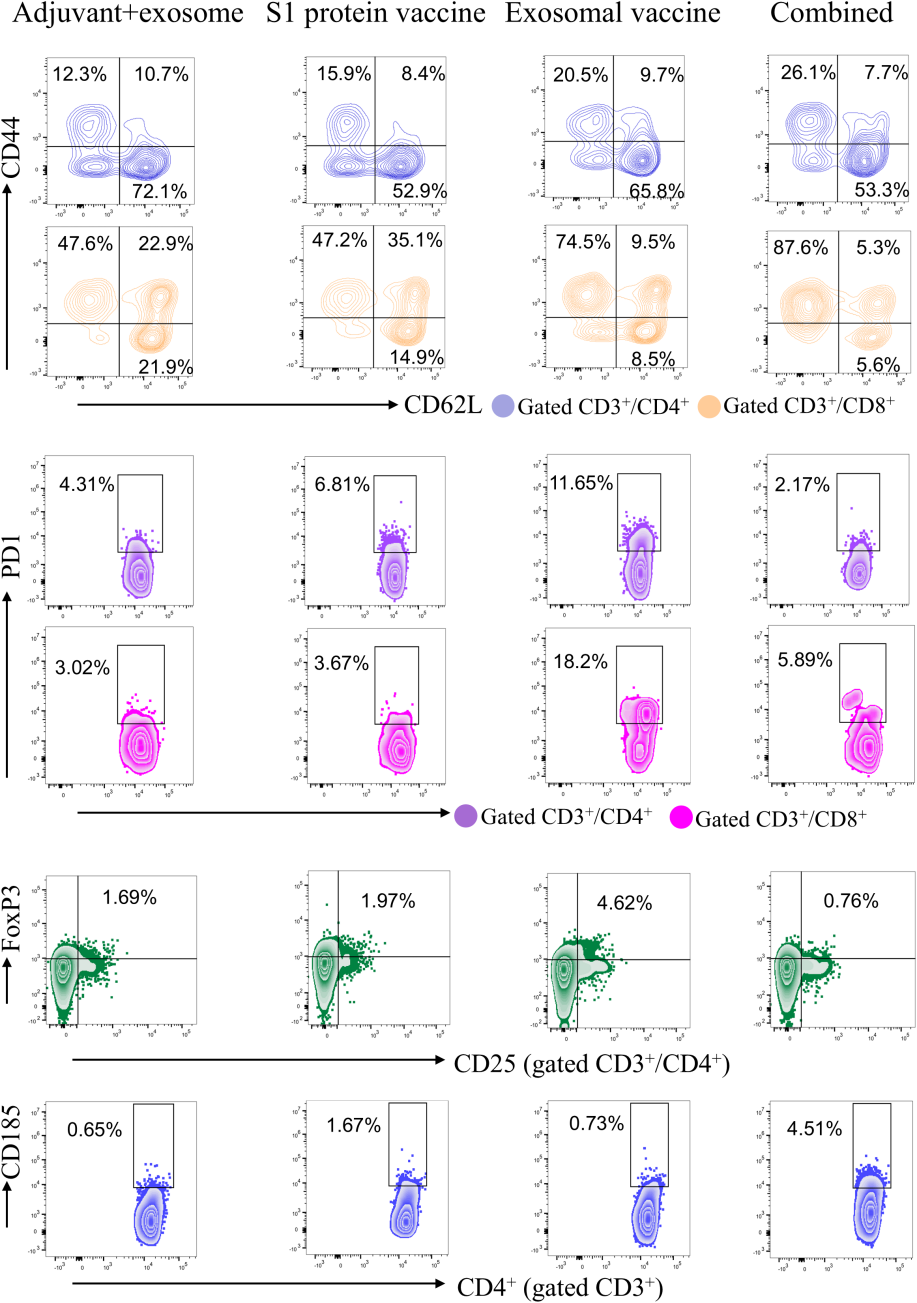
Representative flow cytometric dot plots of T cell phenotype analysis for each immunization group. On day 28 post-immunization, splenocytes from each mouse in each immunization group (containing adjuvant poly I:C) were stained with a series of antibodies and analyzed by multicolor flow cytometry. The frequencies of CD62L^-^/CD44^+^ cells (Tem), CD62L^+^/CD44^+^ cells (Tcm), CD62L^+^/CD44^-^ cells (T naive), and PD-1^+^ cells (Tex) in CD3^+^/CD4^+^ T cell or CD3^+^/CD8^+^ T cell population were calculated. The frequencies of FoxP3^+/^CD25^+^ cells (Treg) and CD185^+^ cells (Tfh) in CD3^+^/CD4^+^ T cell population were also calculated and displayed in each dot plot.

To further analyze the functional phenotype of T cells in the immunized mice, multicolor flow cytometry was performed to define the changes in T cell functional subsets across the four groups. As showed in **Figure 4**, the combined immunization group obtained significantly higher frequencies of effective memory CD4^+^ T cells (Tem, CD62L^-^/CD44^+^), and significantly lower frequencies of exhausted CD4^+^ T cells and CD8^+^ T cells (Tex, PD-1^+^) and regulatory CD4^+^ T cells (Treg, CD25^+^/FoxP3^+^), as compared to the exosomal T-cell epitope vaccine group. Meanwhile, the combined immunization group also displayed significantly higher frequency of CD4^+^ Tfh cells (CD185^+^) than the S1 protein vaccine group. These data indicate that S1 protein vaccine may help the T-cell epitope vaccine enhance memory T cell immunity and decrease the exhausted and inhibitory T cell responses, while the exosomal T-cell epitope vaccine may help S1 protein vaccine increase antibody titer. The representative flow cytometric dot plots of each group are displayed in **Figure 5**. The flow cytometric dot plots of T cell phenotype analysis for each mouse are shown in **Supplementary Figure S4**-**S6**.

### Combined immunization of exosomal vaccine and recombinant S1 protein vaccine elicits more robust humoral immune responses in HLA-A2/DR1 transgenic mice

**Figure 6:**
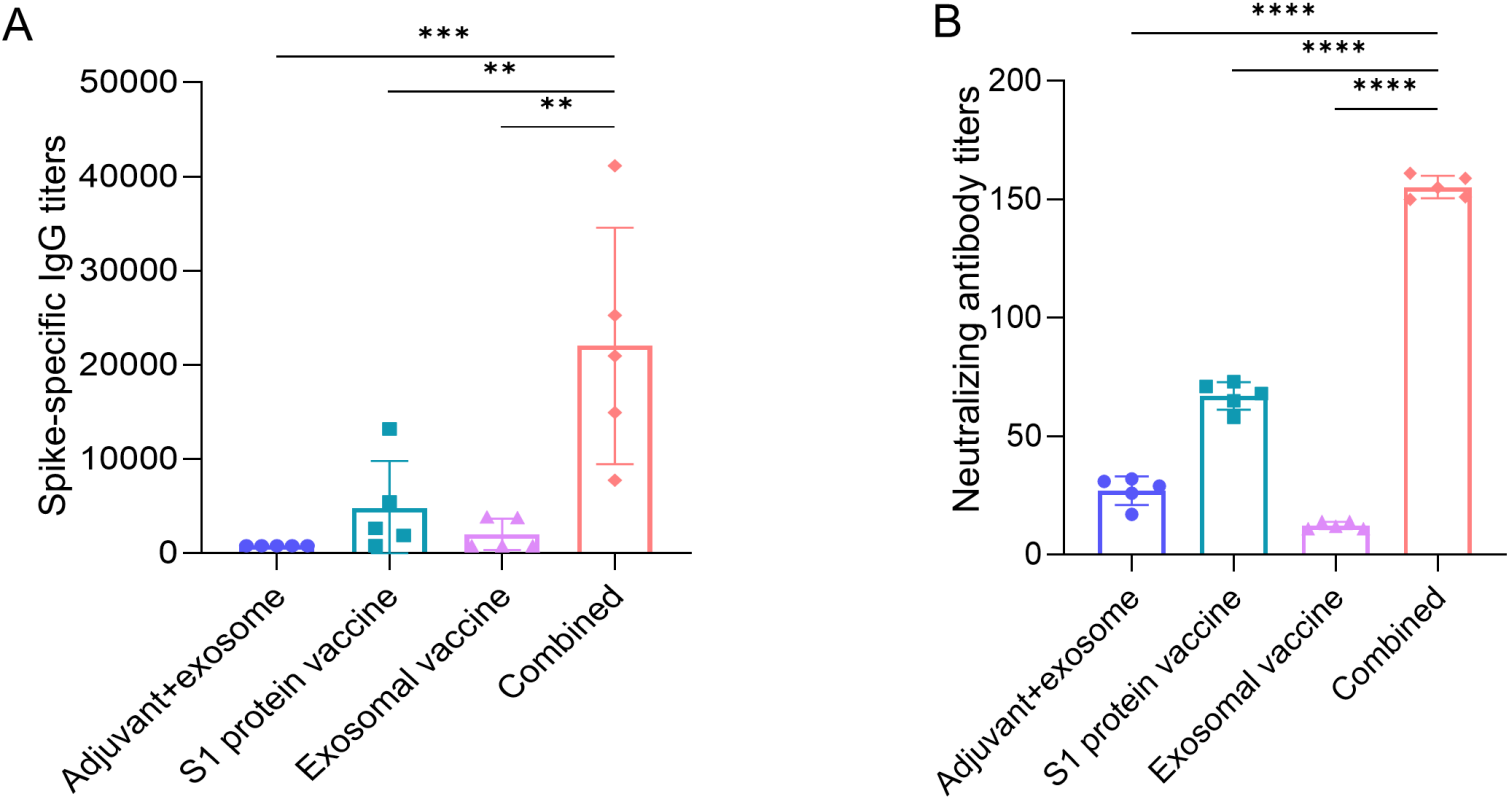
Titer profiles of serum S1 protein-specific IgG antibody and neutralizing antibody in each immunization group. On day 28 post-immunization, serum samples from each mouse in each immunization group (containing adjuvant poly I:C) were analyzed by ELISA for S1 protein-specific IgG antibody titers **(A)** and by BA.5 pseudovirus and HEK-293T-ACE2 cell neutralization experiment for BA.5-targeted neutralizing antibody titers **(B)**. Five HLA-A2/DR1 transgenic mice per group. Mann-Whitney test and Kruskal–Wallis H test were used. ****p<0.0001; ***p<0.001; **p<0.01.

In parallel with cellular immunity assessment, the humoral immunity was also evaluated. Measurement of S1 protein-specific IgG antibody titers (day 28) revealed obviously higher levels in the combination group compared to S1 protein vaccine groups detected by ELISA (**Figure 6A**). Crucially, pseudovirus neutralization assays using HEK-293T-ACE2 cells demonstrated significantly higher neutralizing antibody titers in the combination group than the S1 protein vaccine group (**Figure 6B**), indicating synergistic enhancement of humoral responses, particularly in neutralizing antibody induction. In parallel, exosomal T-cell epitope vaccine group did not elicit humoral immune response as compared to adjuvant+ exosome control group.

### Combined immunization of exosomal T-cell epitope vaccine with recombinant S1 protein vaccine induces synergistic protective efficacy against SARS-CoV-2 infection in HLA-A2/DR1/hACE2 mice

**Figure 7:**
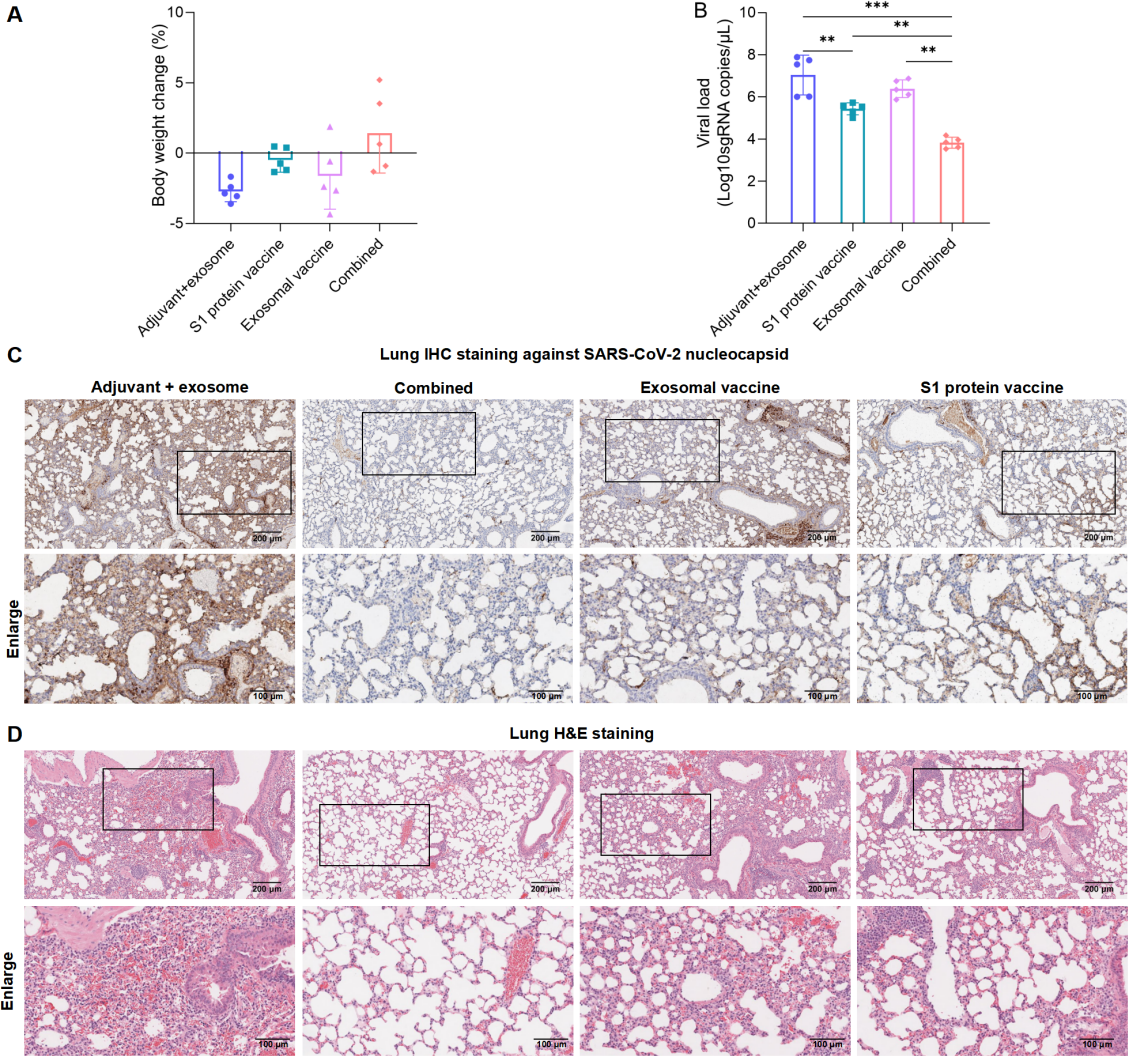
Protective efficacy against SARS-CoV-2 infection after immunization in HLA-A2/DR1/hACE2 transgenic mice. On day 28 post-immunization, each moue in each immunization group (containing adjuvant poly I:C) was challenged with SARS-CoV-2 BA.5 variant. On day 33, the body weight was measured **(A)**, viral loads in pulmonary tissues were quantified by RT-qPCR **(B)** and evaluated by IHC staining to nucleocapsid protein of SARS-CoV-2 **(C)**, and the inflammatory cell filtration and histopathological damage was evaluated by H&E staining in lung lesions **(D)**. Five HLA-A2/DR1/hACE2 transgenic mice per group. Mann-Whitney test and Kruskal–Wallis H test were used. ***p<0.001; **p<0.01; *p<0.05.

HLA-A2/DR1/hACE2 transgenic mice were employed to evaluate the protective efficacy of the combined immunization. On day 28 (7 days after the final booster), mice were challenged with SARS-CoV-2 BA.5 live virus and followed by viral loads quantification and IHC as well as H&E staining in lungs on day 33(5 days after virus challenge). The combined immunization group exhibited weight gain versus weight loss in other three groups (**Figure 7A**), and displayed significantly reduced pulmonary viral loads as quantified by qRT-PCR (**Figure 7B**) and evaluated by IHC staining to nucleocapsid protein of SARS-CoV-2 (**Figure 7C**), and attenuated histopathological damage and inflammatory cell infiltration (**Figure 7D**), when compared to the S1 protein vaccine group or exosomal T-cell epitope vaccine group. These findings demonstrate that the combined immunization of exosomal T-cell epitope vaccine and antibody-inducing vaccine (S1 protein) can achieve synergistic protective efficacy against virus infection through coordinated humoral and cellular immunity, providing strategic insights for COVID-19 vaccine optimization.

## Discussion

Contemporary immunological principles emphasize that synergistic humoral and cellular immunity constitutes the critical antiviral defense mechanism. Nonetheless, antibody-inducing vaccines remain the priority for development in emergency response to new outbreaks. For example, the current licensed vaccines of SARS-CoV-2 are all designed to display or express the S protein, thereby stimulating the body to produce neutralizing antibodies, such as recombinant spike protein vaccine, adenoviral vector vaccine, and mRNA vaccine. This is mainly due to the fact that neutralizing antibodies can rapidly block the virus from invading host cells and quickly establish herd immunity. Meanwhile, the design of antibody-inducing vaccine does not need to consider the restriction of HLA polymorphism in the population, enabling them to have a wide application range. However, in the face of the long-term prevalence of SARS-CoV-2, the titers of neutralizing antibodies induced by antibody-inducing vaccines significantly decline by approximately 70–80% within 6 months, making it difficult to maintain long-term protection (14). Meanwhile, SARS-CoV-2 undergoes frequent mutations, mainly concentrated in the receptor-binding domain (RBD) of the S protein, causing the binding affinity and neutralizing capacity of existing neutralizing antibodies against new variants to decline rapidly (15). Therefore, sole reliance on antibody-inducing vaccines can no longer meet the needs of long-term prevention and control, which has triggered researches on the potential of T-cell epitope vaccines.

Nowadays, some T-cell epitope vaccines of SARS-CoV-2 have entered clinical trials, but the data from clinical combined use with antibody-inducing vaccine is still very limited. Building upon our previous researches on RBC-derived exosome and exosomal vaccine loading multipeptides of SARS-CoV-2 (13), this study systematically evaluated the synergistic immune responses and protective efficacy induced by the combined immunization of recombinant S1 protein vaccine and exosomal T-cell epitope vaccine carrying 19 CD4^+^ T-cell epitope peptides and 27 CD8^+^ T-cell epitope peptides in the highly humanized mice. Our data highlight the complementary value of T-cell epitope vaccine for antibody-inducing vaccine. The combined immunization obviously induced a synergistic increase of S1 protein-specific IgG antibodies, neutralizing antibodies and Tfh cells as compared to the S1 protein vaccine alone. Meanwhile, the combined immunization induced significantly more S1 protein-specific T cells as compared to the exosomal T-cell epitope vaccine alone, but had no enhanced effect on specific T cells against other proteins, which is in line with the expectation of the antigen specificity of the S1 protein vaccine. More importantly, the S1 protein vaccine helped the exosomal T-cell epitope vaccine significantly increase the frequency of overall effector memory CD4^+^ T cells and significantly inhibit the differentiation of T cells into exhausted and regulatory T cells. In the viral challenge, the combined immunization also achieved the strongest protective efficacy as compared to the mono-vaccine immunization groups, including reduced viral loads, pulmonary inflammatory cell infiltration, and histopathological damage. The detailed synergistic mechanisms therein deserve further investigation, as does their impact on the lasting immune protection. The potential underlying factors can be summarized as follows: (i) shared epitope motifs between S1 protein and multipeptide vaccine, (ii) exosome-mediated dendritic cell activation surpassing adjuvant effects, (iii) Tfh cell-driven germinal center B cell maturation, and (iv) enhanced antigen cross-presentation through exosomal epitope delivery, collectively establishing a coordinated defense network against viral infection.

Another advantage of this study is the utilization of RBC-derived exosomes as a carrier/deliverer of multipeptide vaccine depending on an in-house invented loading method. For the current undergoing T-cell epitope vaccines of SARS-CoV-2, multipeptides are directly mixed with adjuvant, or delivered by gold-nanoparticles (6, 8, 9, 16). No exosomes were used as the deliverer of SARS-CoV-2 T-cell epitope peptides. Exosomes are small extracellular vesicle with a diameter of 40–150 nm which can be secreted by all types of cells. They carry cargos like protein, RNA and lipids (17) and participate in cell-cell communication (18). Thus, people have gradually recognized its potential as a new platform for vaccine delivery. Exosomal vaccines have been broadly applied to anti-infection and anti-cancer therapy (19), and some have even entered clinical trials (19). Apart from the advantages of good biocompatibility, low immunogenicity, low toxicity, high stability during blood circulation or in high-temperature storage and transportation environments, and easy to engineer, exosomes are also capable of cross-presenting exogenous protein antigens, thereby inducing CD8^+^ T cell immune responses (20). Furthermore, the cellular components carried by exosomes themselves may also be involved in the immune activation process as adjuvant. Here, the exosomes were purified from red blood cells as carriers for T-cell epitope peptides based on three main reasons. First, RBCs are abundant in source and their exosomes can be mass-produced in large quantities for future use. Second, as anucleated cells, RBCs do not express HLA molecules, resulting in minimal immune rejection during allogeneic application. Third, although RBC-derived exosomes may carry ABO blood group antigens, the pre-existing anti-A or anti-B antibodies in recipient are naturally occurring pentameric IgM antibodies. Due to their large molecular weight, these antibodies are predominantly confined to the vascular system. Given that the exosomal T-cell epitope vaccine in our study was administered via subcutaneous injection, the exosomes are unlikely to encounter and bind to the pre-existing ABO blood group antibodies in the recipient, thus preventing exosomes damage. As introduced in the systematic review and meta-analysis of clinical trials, the human extracellular vesicle-based therapy is safe and low in immunogenicity, with a low incidence of serious adverse events (SAE; 0.7% (95%-CI: 0.1–5.2%) and adverse events (AE; 4.4% (95%-CI: 0.7–22.2%). Subgroup analysis showed no significant difference in SAE between autologous and allogeneic administration, as well as between engineered and non-engineered extracellular vesicle products (21).

In another hand, although many approaches have been developed to introduce proteins or peptides into exosomes (22), most methods need the exosome surface or payload pre-modification. Loading multiple peptides or proteins into exosomes remains a challenge. In our previous research, a novel self-assembly approach to load proteins or peptides of interest into the exosome membrane was developed without payload or exosome pre-modifications (13), and was utilized in this study. More importantly, our previous researches have demonstrated that RBC-derived exosomes loading CD8^+^ T-cell epitope peptides of SARS-CoV-2 can elicit much more stronger epitope-specific CD8^+^ T cell responses than the multipeptides mixed with adjuvant in the HLA-A2/DR1 transgenic mice (11, 13).

## Conclusions

In conclusion, this study performed the combined administration of exosomal T-cell epitope vaccine and S1 protein vaccine, and systematically evaluated the specific T cell response and neutralizing antibody production in the HLA-A2/DR1 transgenic mice and the protective efficacy in the HLA-A2/DR1/hACE2 transgenic mice. Our findings underscore the synergistic benefits of activating both humoral and cellular immunity and the enhanced protective efficacy against SARS-CoV-2 infection, outperforming mono-vaccine regimen. This study provide the critical preclinical evidence from highly humanized mice for optimizing next-generation SARS-CoV-2 vaccine protocols.

## Data Availability

The original contributions presented in the study are included in the article/supplementary material. Further inquiries can be directed to the corresponding authors.
