# Supplementary material for: Synergistic immune protection of exosomal T-cell epitope vaccine and antibody-inducing vaccine against SARS-CoV-2 in highly humanized mice
Source: Front Immunol. 2026 Feb 3;17:1729444. doi: 10.3389/fimmu.2026.1729444 (PMC12909587; doi:10.3389/fimmu.2026.1729444)
Supplement: Supplementary file 1 [file DataSheet1.pdf]

Synergistic immune protection of exosomal T-cell epitope vaccine and antibody-inducing vaccine against SARS-CoV-2 in highly humanized mice

Anran Shen, Suyue Zhu, Min Li, Yu Zhao, Yandan Wu, Yue Zhang, Jiejie Zhang, Xuelian Han, Yuan Wang, Guangyu Zhao, Linli Lv, Qi Yin, Taotao Tang

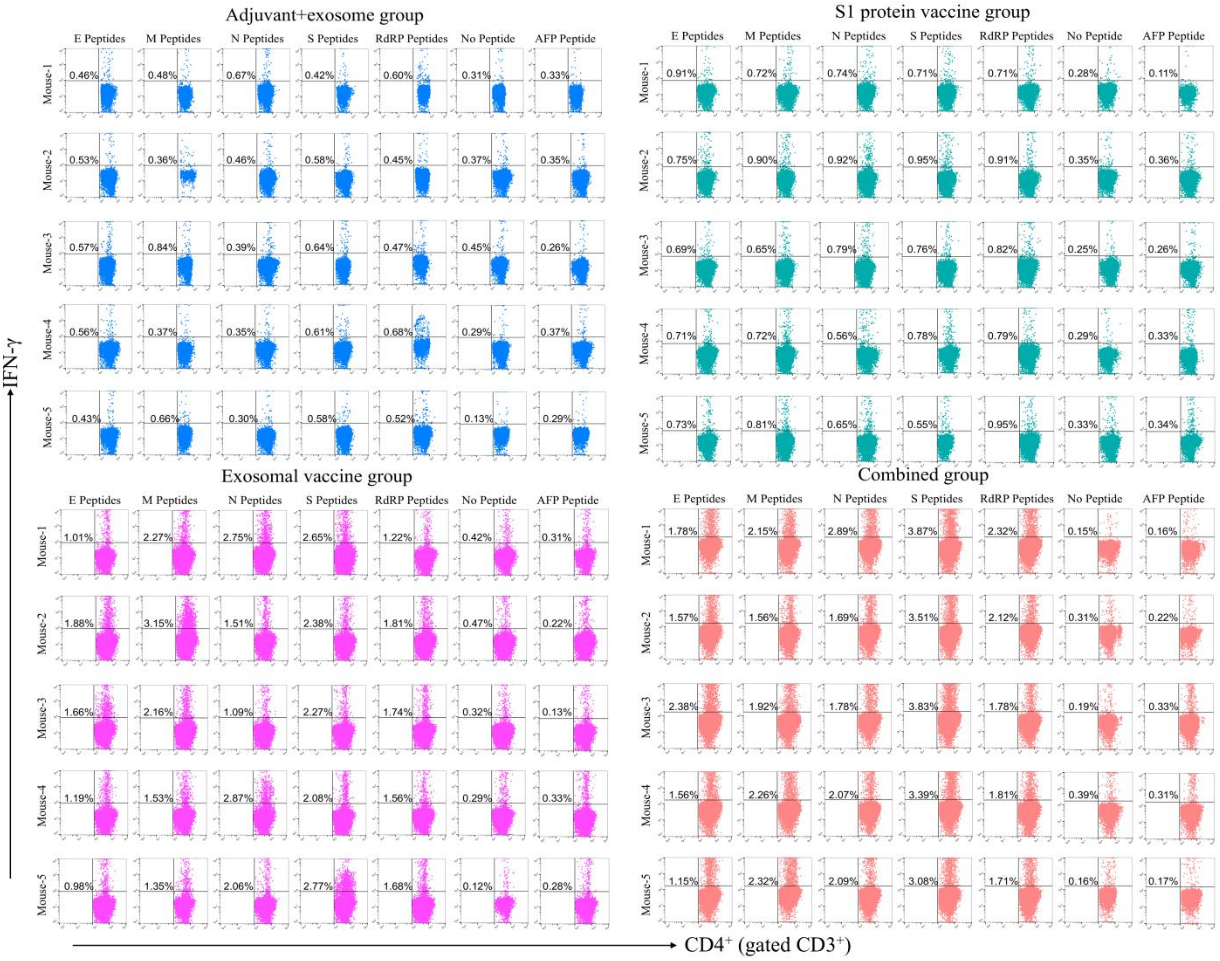

Figure S1 Flow cytometric dot plots of epitope-specific CD4$^+$ T cells for each mouse in each immunization group

On day 28 post-immunization, splenocytes from each mouse in each immunization group were *ex vivo* co-cultured with 5 peptide pools (19 CD4$^+$ T-cell epitopes derived from E, M, N, S, and RdRp proteins and used to immunize mice) for 22 hours followed by IFN-γ ICS assays. The frequencies of IFN-γ^+/CD4$^+$ T cells reactive to the epitope peptides derived from each protein in each mouse were displayed in the upper-left quadrant of each dot plot. No peptide: splenocytes alone well, negative control in ICS assay; AFP peptide: irrelative peptide (HLA-DR1-restricted $AFP_{421-436}$ peptide) well, negative control in ICS assay.

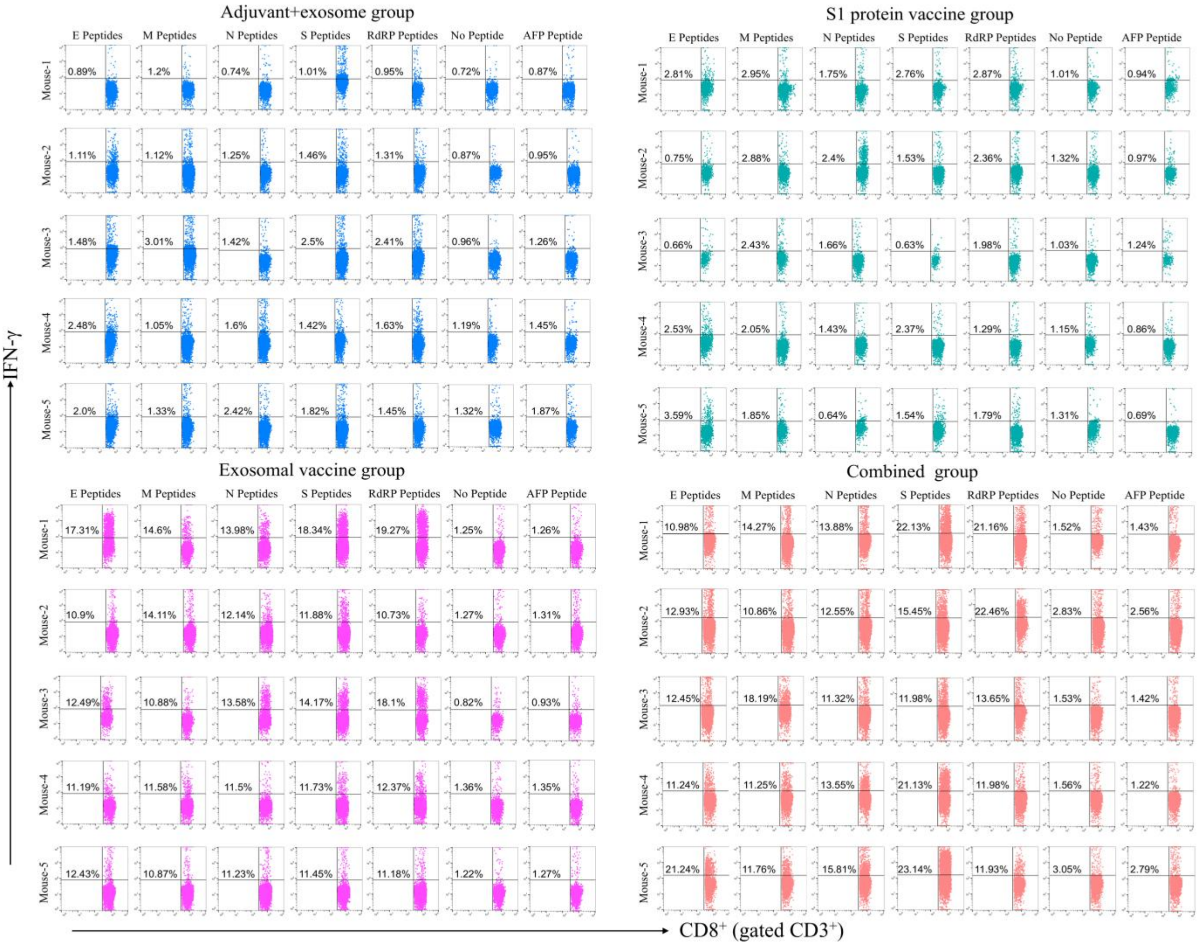

Figure S2 Flow cytometric dot plots of epitope-specific $CD8^+$ T cells for each mouse in each immunization group

On day 28 post-immunization, splenocytes from each mouse in each immunization group were *ex vivo* co-cultured with 5 peptide pools (27 $CD8^+$ T-cell epitopes derived from E, M, N, S, and RdRp proteins and used to immunize mice) for 22 hours followed by IFN-γ ICS assays. The frequencies of IFN-γ^+/$CD8^+$ T cells reactive to the epitope peptides derived from each protein in each mouse were displayed in the upper-left quadrant of each dot plot. No peptide: splenocytes alone well, negative control in ICS assay; AFP peptide: irrelative peptide (HLA-A2-restricted $AFP_{158-166}$ peptide) well, negative control in ICS assay.

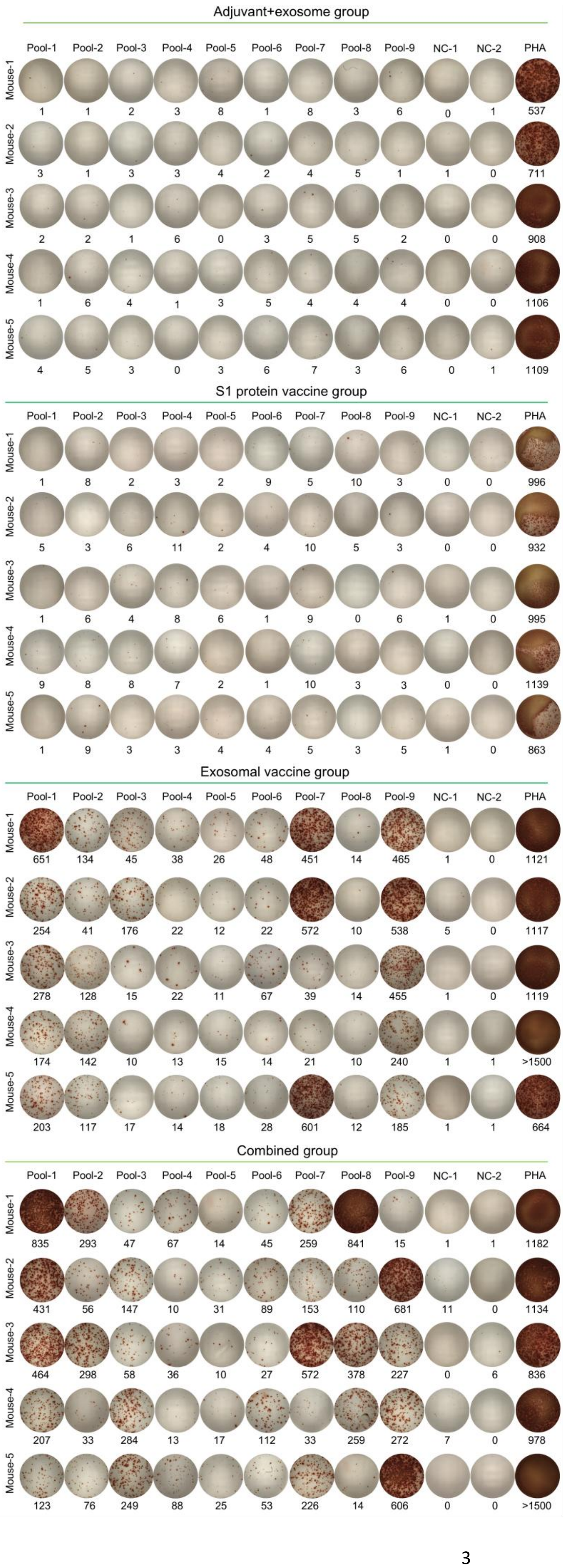

Figure S3 Dot plots of epitope-specific T cells for each mouse in each immunization group as detected by ELISpot assays

On day 28 post-immunization, splenocytes from each mouse in each group were *ex vivo* co-cultured with 9 peptide pools (27 $CD8^+$ T-cell epitopes and 19 $CD4^+$ T cell epitopes used to immunize mice) for 20 hours followed by IFN-γ ELISpot assay. The SFUs of T cells reactive to each peptide pool were displayed below the each dot plot image. NC1: splenocytes alone well, negative control in ELISpot assay; NC2: irrelative peptides ($AFP_{158-166}$ and $AFP_{421-436}$ peptides) well, negative control in ELISpot assay; PHA: positive control well in ELISpot assay.

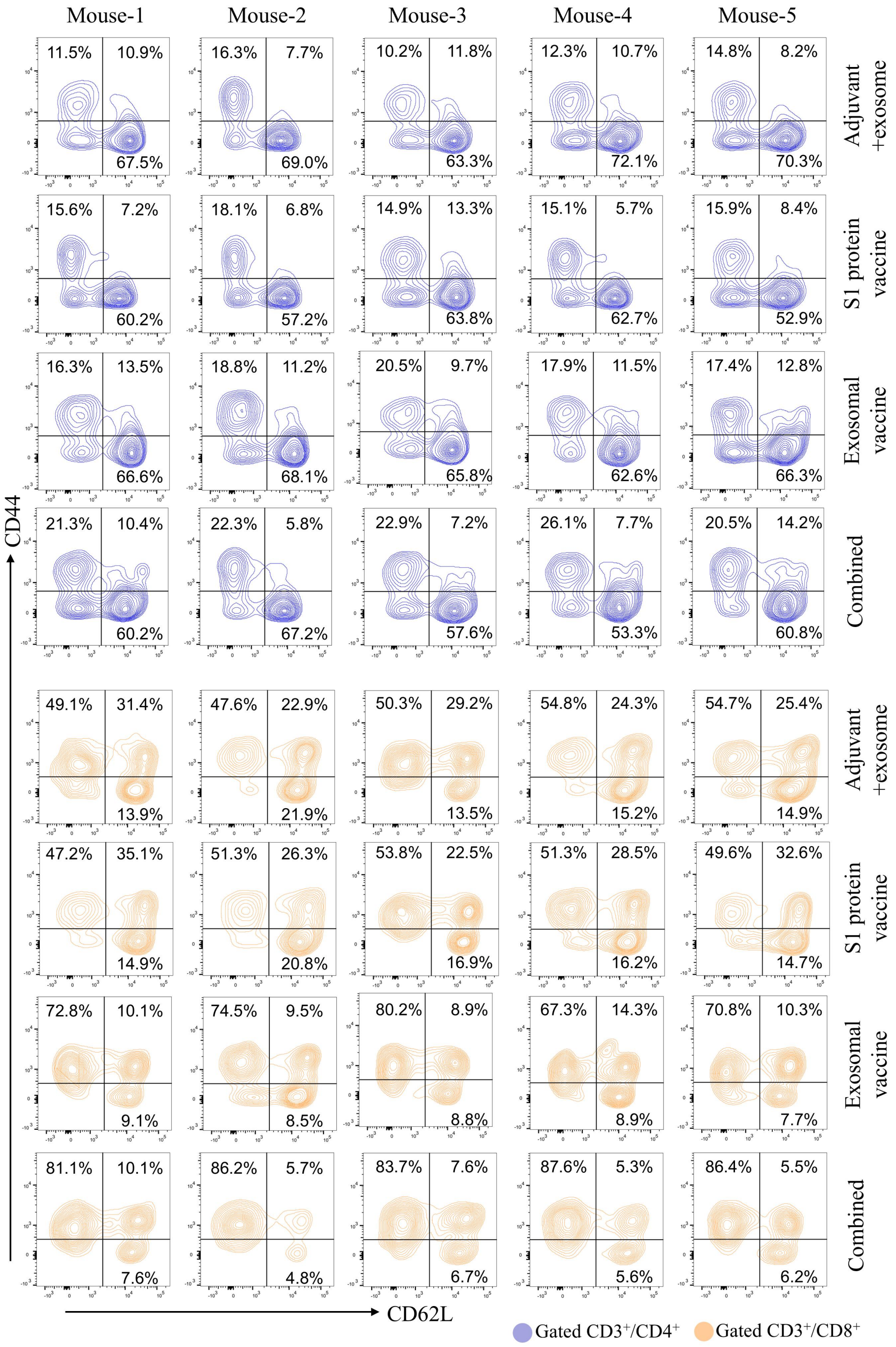

Figure S4 Flow cytometric dot plots of T cell phenotype analysis for each mouse in each immunization group

On day 28 post-immunization, splenocytes from each mouse in each group were stained with CD44, CD62L, CD3, and CD4 or CD8 antibodies and analyzed by multicolor flow cytometry. The frequencies of $CD62L^-/CD44^+$ cells (Tem), $CD62L^+/CD44^+$ cells (Tcm), and $CD62L^+/CD44^-$ cells (T naive) in $CD3^+/CD4^+$ T cell population or $CD3^+/CD8^+$ T cell population were calculated and displayed in the corresponding quadrant of each dot plot.

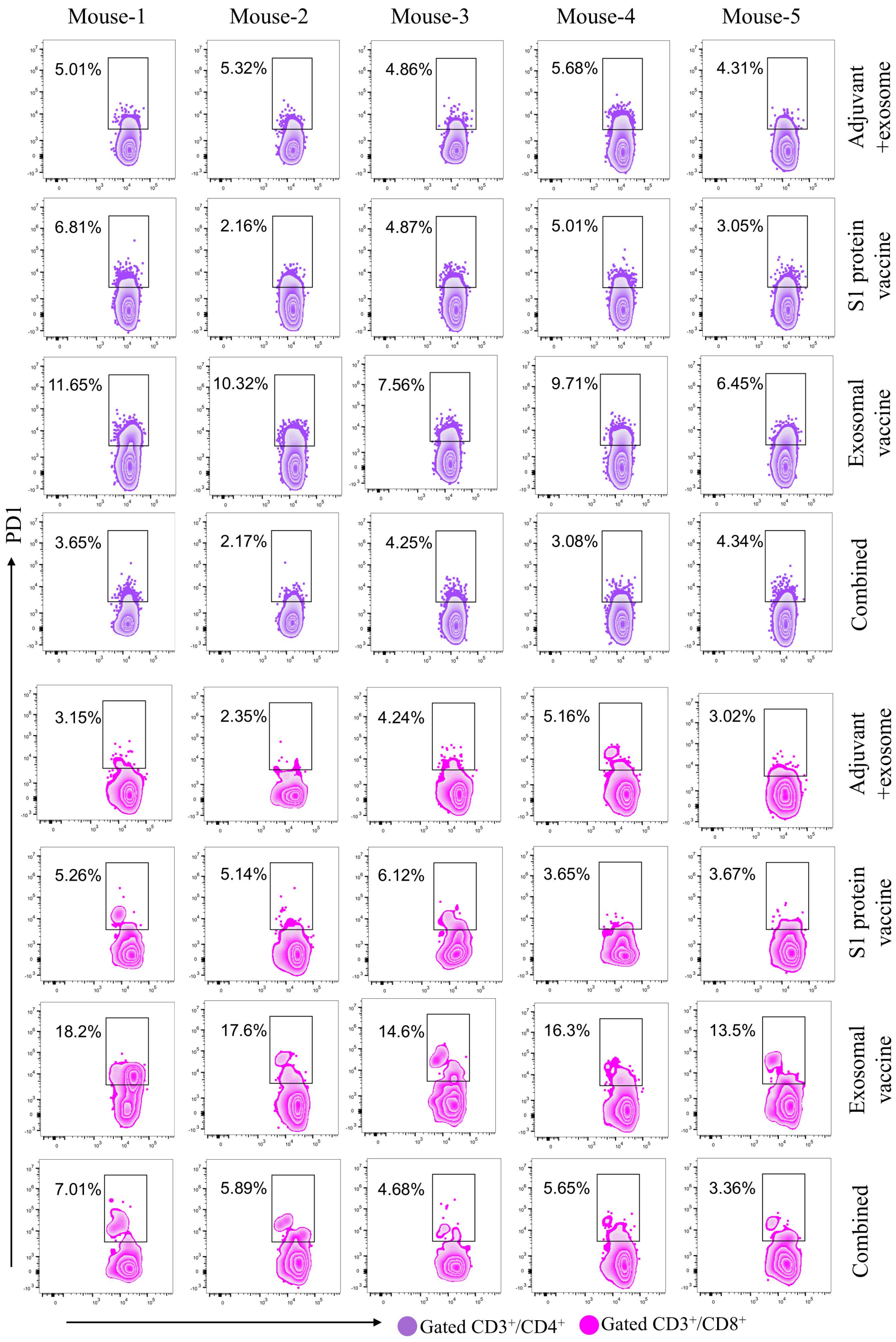

Figure S5 Flow cytometric dot plots of T cell phenotype analysis for each mouse in each immunization group

On day 28 post-immunization, splenocytes from each mouse in each immunization group were stained with PD-1, CD3, and CD4 or CD8 antibodies and analyzed by multicolor flow cytometry. The frequencies of PD-1$^+$ cells in CD3$^+$/CD4$^+$ T cell population (CD4$^+$ Tex) or CD3$^+$/CD8$^+$ T cell population (CD8$^+$ Tex) were calculated and displayed in each dot plot.

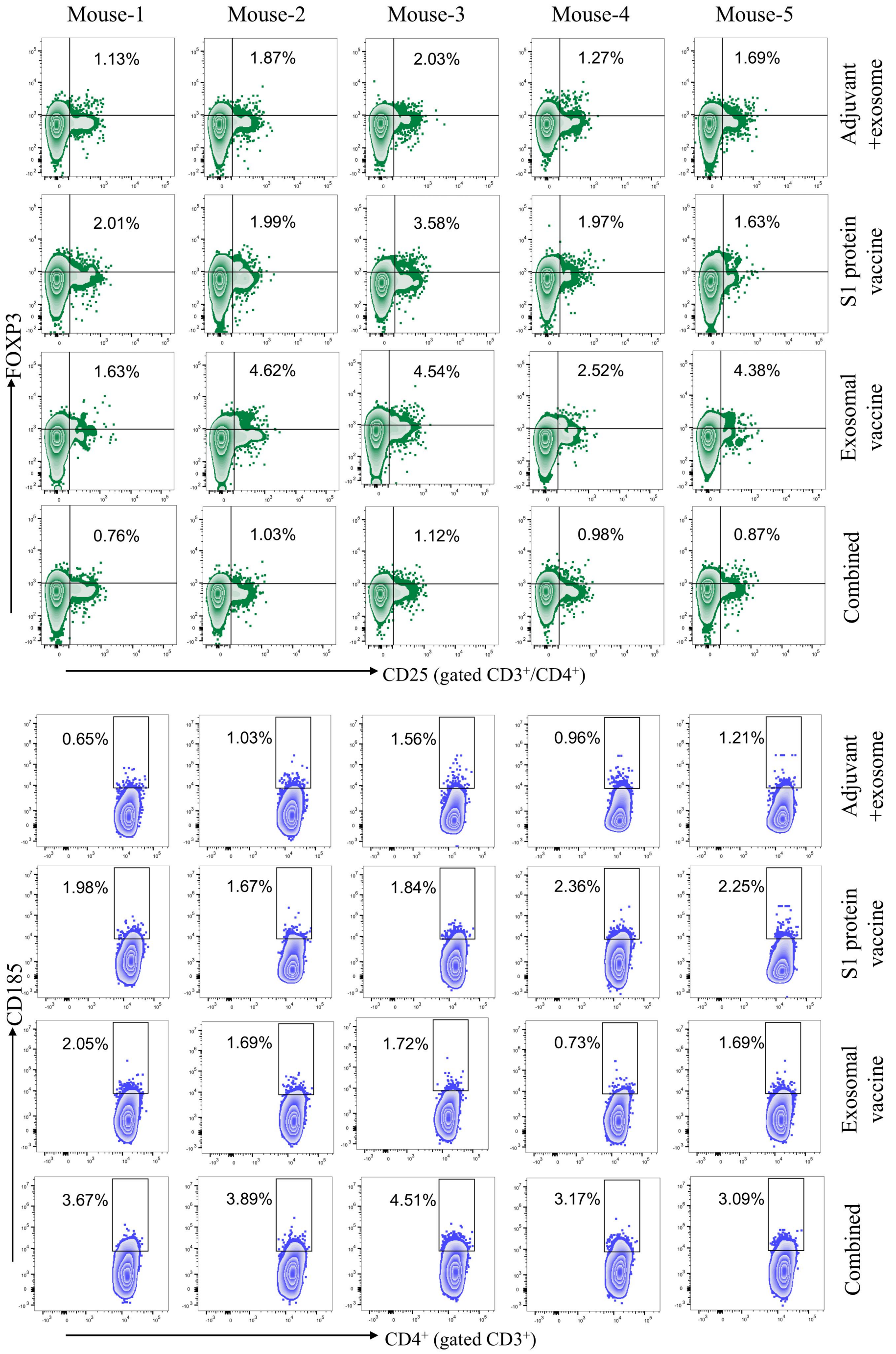

Figure S6 Flow cytometric dot plots of T cell phenotype analysis for each mouse in each immunization group

On day 28 post-immunization, splenocytes from each mouse in each immunization group were stained with FoxP3, CD25, CD3 and CD4 antibodies, or stained with CD185,CD3 and CD4 antibodies, and analyzed by multicolor flow cytometry. The frequencies of FoxP3^{+}/CD25^{+} cells in CD3^{+}/CD4^{+} T cell population (CD4^{+} Treg) or CD185^{+} cells in CD3^{+}/CD4^{+} T cell population (CD4^{+} Tfh) were calculated and displayed in each dot plot.
